# Wide distribution of prion infectivity in the peripheral tissues of vCJD and sCJD patients

**DOI:** 10.1007/s00401-021-02270-x

**Published:** 2021-02-02

**Authors:** Jean-Yves Douet, Alvina Huor, Hervé Cassard, Séverine Lugan, Naima Aron, Mark Arnold, Didier Vilette, Juan-Maria Torres, James W. Ironside, Olivier Andreoletti

**Affiliations:** 1 UMR INRA ENVT 1225, Interactions Hôtes Agents Pathogènes, Ecole Nationale Vétérinaire de Toulouse, 23 Chemin des Capelles, 31076 Toulouse, France; 2 APHA Sutton Bonington, Loughborough, LE12 5NB Leicestershire UK; 3 Centro de Investigación en Sanidad Animal, CISA-INIA, Valdeolmos, Spain; 4 Centre for Clinical Brain Sciences, University of Edinburgh, Western General Hospital, Edinburgh, EH4 2XU UK

## Abstract

Sporadic Creutzfeldt-Jakob disease (sCJD) is the commonest human prion disease, occurring most likely as the consequence of spontaneous formation of abnormal prion protein in the central nervous system (CNS). Variant Creutzfeldt–Jakob disease (vCJD) is an acquired prion disease that was first identified in 1996. In marked contrast to vCJD, previous investigations in sCJD revealed either inconsistent levels or an absence of PrP^Sc^ in peripheral tissues. These findings contributed to the consensus that risks of transmitting sCJD as a consequence of non-CNS invasive clinical procedures were low. In this study, we systematically measured prion infectivity levels in CNS and peripheral tissues collected from vCJD and sCJD patients. Unexpectedly, prion infectivity was detected in a wide variety of peripheral tissues in sCJD cases. Although the sCJD infectivity levels varied unpredictably in the tissues sampled and between patients, these findings could impact on our perception of the possible transmission risks associated with sCJD.

**Supplementary Information:**

The online version contains supplementary material available at 10.1007/s00401-021-02270-x.

## Introduction

Transmissible spongiform encephalopathies (TSEs), or prion diseases, are fatal neurodegenerative disorders that affect a large spectrum of mammalian species. The fundamental event in prion propagation is the conversion of the normal cellular prion protein (PrP^C^) into an abnormal disease-associated isoform (PrP^Sc^) in the brain and other tissues of infected individuals. PrP^C^ is completely degraded by digestion with proteinase K (PK), whereas PrP^Sc^ is N-terminally truncated, resulting in a PK-resistant core termed PrP^res^ [41]. According to the prion concept, PrP^Sc^ is the principal, if not the sole, component of the transmissible prion agent [49] and PrP^res^ is a tissue marker for prion diseases [41, 51]. Particular biochemical properties of PrP^Sc^, such as detergent solubility, PK resistance and electromobility evidenced by western blot can be used to distinguish between different prion agents or strains [5, 6]

In humans, Creutzfeldt-Jakob disease (CJD) is a low incidence disease (≈ 1 case per million population per year) that occurs most often as either a sporadic (sCJD) or a familial/genetic (fCJD) form. Whereas familial disease forms are linked to pathogenic mutations in the human prion protein gene (*PRNP*), no clear epidemiologic risk factors have been identified for sporadic disease forms [57]. In the absence of identifiable external sources that might explain sCJD occurrence, it is currently assumed that this disease is triggered by the spontaneous and stochastic formation of a misfolded PrP nucleus in the brain of the affected individual. This original nucleus is considered to recruit and convert fresh PrP^C^ into PrP^Sc^ leading to the progressive spread and propagation of prions in the brain of the diseased patient. In fCJD cases, *PRNP* mutations foster the formation of the initial misfolded PrP nucleus, leading to an increased probability of development of the disease [14].

In sCJD, two major brain PrP^res^ isoforms have been described by western blot (WB): in type 1 PrP^res^, the apparent molecular weight of the unglycosylated fragment is 21 kDa, while in type 2, it is 19 kDa [45]. Sporadic CJD is not a uniform disorder in terms of its clinical and neuropathological phenotypes and is subclassified according to the polymorphism at codon 129 of the *PRNP* sequence (Methionine/Valine) and to the electromobility of the PK resistant core of the abnormal PrP (PrP^res^: either as type 1 or 2) [45]. The experimental transmission of sCJD tissue isolates in transgenic mice that express human PrP (TgHu) has confirmed that sCJD is associated with a several prion strains that vary in their biological and biochemical properties [7, 11]. Although the complete spectrum of sCJD-associated prion strain remains unknown, there is now significant evidence that the two most frequent categories of sCJD, MM/MV type 1 and MV/VV type 2, are predominantly caused by two distinct prion strains, named M1^CJD^ and V2^CJD^ respectively [7, 11, 12, 25, 33].

In 1996, a new form of CJD, named variant CJD (vCJD), was identified in humans. Variant CJD was demonstrated to be caused by the prion strain responsible for bovine spongiform encephalopathy in cattle, which is distinct from any of the identified sCJD prion strains [29, 30]. Following the emergence of vCJD, it was established that in both affected and asymptomatic patients the vCJD agent accumulated in a large variety of peripheral tissues, including lymphoid organs and blood [15]. On the basis of these findings, the risk of vCJD transmission by surgical and medical procedures has been considered as serious by international health authorities, leading to the implementation of systematic preventive measures aiming at limiting the risk of iatrogenic vCJD transmission [56].

In marked contrast with vCJD, previous investigations carried out in sCJD patients revealed either low or inconsistent levels or an absence of detectable PrP^Sc^ in peripheral tissues [24, 28]. These findings led to the general consensus that the risk of transmitting sCJD in the framework of a non-Central Nervous System (CNS) invasive clinical procedure was low.

Recently, bioassays in transgenic mice that express the human PrP gene and display a high sensitivity to sCJD demonstrated the presence of prion infectivity in the plasma and bone marrow of several sCJD patients [17, 32]. These results raised questions about the overall distribution of prion infectivity and transmission risks associated with other peripheral tissues from sCJD patients.

In this study, we measured the prion infectivity levels in a panel of tissues collected from vCJD and sCJD MM1 cases. These bioassays demonstrated that, as expected, consistent titres of infectivity were present in lymphoid tissues from vCJD patients. However, for the non-lymphoid peripheral tissues studied, variable and lower titres of infectivity were detected in both sCJD and vCJD patients. These findings could impact on our perception of the possible transmission risks associated with sCJD involving non-CNS invasive procedures.

## Methods

### Ethics statement

All animal experiments were performed in compliance with institutional and French national guidelines and in accordance with the European Community Council Directive 86/609/EEC. The animal experiments that are part of this study (national registration 01734.01) were approved by the local ENVT ethic committee. Mouse inoculations were performed under anaesthesia (isofulorane). Mice that displayed clinical signs were anesthetized with isoflurane before sacrifice using CO_2_ inhalation.

Human tissue samples were obtained from the National CJD Research & Surveillance Unit Brain and Tissue Bank in Edinburgh, UK, which is part of the Edinburgh Brain and Tissue Bank. For the purposes of this study, samples were pseudo-anonymized using a Brain Bank reference number. All cases had informed consent for research and their supply and use in this study in keeping with the East of Scotland Research Ethics Service approval for the Edinburgh Brain and Tissue Bank (16/ES/0084).

### CJD and control patients

Tissues from four clinical vCJD cases (referred to in this study as vCJD-1 to vCJD-4) and one asymptomatic vCJD-infected individual who received a transfusion of packed red blood cells from a donor who subsequently died from vCJD [47] (vCJD-AS) were investigated. The vCJD cases were homozygous for methionine at codon 129 (Met/Met_129_), while the asymptomatic case was heterozygous Methionine/Valine at this particular codon. Similarly, tissues from five sCJD cases were investigated. Cases were selected on the basis of their genotype at codon 129 (Met/Met_129_), their PrP^res^ Western blot profile (type 1) and availability of peripheral tissues samples. Tissues from one non-CJD case (Met/Met_129_) were used as control (NC-1). The autopsies were performed in various hospitals across the UK with appropriate facilities for cases of suspected CJD. For this group, the median post mortem interval was 48 h (range 30–72 h).

Basic demographic and medical history details for the vCJD, sCJD and control cases are presented in Table 1. In all cases, informed consent was given to examine the entire *PRNP* coding sequence in order to exclude pathogenic mutations in the *PRNP* gene [2, 42].

**Table 1:** Clinico-pathological data and medical histories of sCJD and vCJD cases whose tissues were tested by bioassay

Patient	*PRNP* genotype codon 129	Gender	Age at onset (years)	Disease duration^a^	Medical and surgical history
sCJD 1	MM	F	73	3 mo	Tonsillectomy (age 6 y), idiopathic thrombocytopenia purpura (age 31 y), splenectomy (age 33 y), thyroidectomy (age 51 y)
sCJD 2	MM	F	57	24 mo	Hysterectomy (age early 30 s), breast cancer lumpectomy (age 46 y), mastectomy (age 53 y), axillary node clearance (age 54 y)
sCJD 3	MM	M	67	1 mo	Sutures to hand (age 35 y), excision of cyst on neck (age 45 y), laser treatment to eyes (age 65 y)
sCJD 4	MM	F	65	4 mo	Tonsillectomy (age 20 s), caesarean section (age 35 y), cervical laminectomy and bone graft (age 48y), endometrial polyp excision (age 56 y), bilateral varicose vein surgery (age 61 y), hiatus hernia (age 65 y)
sCJD 5	MM	F	64	3 mo	Bilateral otosclerosis (age 39 y), osteoporosis (age 50 y), gallstones (age 58 y), wound exploration and repair of radial distal nerve + sutures (age 59 y), polypectomy at colonoscopy (age 60 y), closed fracture calcaneus (age 60 y)
vCJD 1	MM	M	25	10 mo	Fractured L medial malleolus (age 11 y), sutures to R thumb (age 18 y)
vCJD 2	MM	M	25	10 mo	Tonsillectomy (age 12 y), fractured L wrist (age 12 y), minor head injury (age 22 y)
vCJD 3	MM	M	32	18 mo	Nasal manipulation (age 14 y), plaque psoriasis (age 27 y)
vCJD 4	MM	F	16	18 mo	Adenoidectomy and insertion of L grommet (age 7 y)
vCJD-AS	MV	F	82	–	Blood transfusion after bowel resection (age 78 y), hyperthyroidism (age 30 s), kidney stones (age 43), hysterectomy (age 45 y), bowel abscess (ag e 49 y), hypothyroidism (age 57 y), chronic obstructive airways disease (age 63 y), excision basal cell carcinoma (age 71y), diverticular disease—> sigmoid resection (age 72 y), congestive cardiac failure (age 74 y), abdominal aortic aneurysm (age 74 y), spinal osteoporosis (age 74 y), acne rosacea (age 74 y), angina pectoralis (age 76 y), cataract operation × 2 (age 79 y and 82 y)
NC	MM	F	80	–	Alzheimer’s disease, cerebral infarction and ischaemia. (No other data available)

^a^Number of months (mo) after disease onset

### Tissues samples collection and tissue homogenate preparation

Tables 2 and 3 shows the tissue tested in each patient. Tissues samples were collected post mortem using disposable equipment, snap frozen, and stored at − 80 °C. Strict precautions were undertaken to prevent potential cross contamination of samples during collection, handling and storage. Frozen samples were homogenized in 5% glucose in distilled water in grinding tubes (Bio-Rad) adjusted to 10% (w/v) using a TeSeE^™^ Precess 48^™^ homogenizer (Bio-Rad). Homogenates were then filtered through a 20 g needle with a syringe.

### Mouse bioassays

vCJD and sCJD tissues bioassay were carried out using mice expressing bovine PrP (tgBov-tg110) and human PrP methionine at codon 129 (tg Met-tg340), respectively. TgMet and tgBov mice expressed approximately fourfold and eightfold more PrP compared to that seen in normal human and cattle brain tissue [12, 13, 44]. These two models were already used to measure prion infectivity in vCJD and sCJD MM1 tissues in previous studies [12, 15, 32].

Tissues from the non-CJD control case were inoculated in both tgBov and tg340 mice.

Groups of 6- to 10-week-old female mice (*n* = 6) were anesthetized and inoculated with 20 µL of a 10% tissue homogenate in the right parietal lobe using a 25-gauge disposable hypodermic needle. Mice were observed daily and their neurological status was assessed weekly. When clinically progressive TSE disease was evident, the animals were euthanized and their brains harvested. Half of the brain from those animals that had displayed TSE clinical signs was fixed by immersion in 10% formol saline and the other half was frozen at – 20 °C. Tissues from animals found dead were frozen (no formalin fixation was performed). In animals where no clinical signs were observed, mice were killed at the end of their natural life-span (600–750 days). In those cases, survival times reported in the table as > 650 dpi or > 750 dpi.

### Infectious prion titre estimates

The infectious titre in a reference 10% weight/vol frontal cortex homogenate from a clinical vCJD case and sCJD cases were established by endpoint titration (intracerebral route) in tgBov and tgMet mice respectively. The infectious titre (LD_50_/g IC in corresponding mice lines) were estimated by the Spearman–Kärber method [40].

The titre of prion infectivity in vCJD and sCJD case samples were estimated using the method developed by Arnold et al*.* [4].

### Abnormal PrP Western blot (WB) detection

PK resistant abnormal PrP extraction (PrP^res^) and Western blot were performed on tissues as previously described [35]. Immunodetection was performed using one Sha31 (1 µg/ml) monoclonal PrP-specific antibody, which recognizes the amino acid sequences YEDRYYRE (145–152) [18].

### Vacuolar lesion profiles

Haematoxylin–Eosin stained paraffin embedded brain tissue sections were used to establish standardised vacuolar lesion profiles in mice as previously described [20, 21]. Each lesion profile was based on data obtained from a minimum of 3 animals.

## Results

### vCJD transmission

Four clinical vCJD cases (Met_129_ homozygous) and one asymptomatic vCJD case (Met_129_/Val_129_ heterozygous) were selected on the basis of their clinico-pathological features and PrP^res^ Western Blot profile in the brain (Table 1).

A panel of frozen tissues that included CNS (frontal cortex), and 14 different peripheral tissues (such as primary and secondary lymphoid tissues, endocrine and exocrine glands, gonads, kidney, lung, liver, heart and skeletal muscles) from each of these 5 cases was constituted (Table 2). Each sample (10% tissue homogenates) was inoculated by the intracerebral route (IC) to bovine PrP expressing mice (tgBov *n* = 6 per sample, 20 µL per mouse); a bioassay model identified in previous studies as a sensitive and robust approach for the detection and the quantification of vCJD infectivity [15, 17].

**Table 2:** Brain and peripheral tissue samples bioassay results in bovine PrP–expressing mice (tgBov) for clinical and asymptomatic vCJD cases

	vCJD-1 (01/76)			vCJD- 2 (00/101)			vCJD- 3 (99/129)			vCJD- 4 (00/25)			vCJD-AS (26/2004)			Negative control	
Tissue	n/n0	Suriv. time	Inf. tit estimates	n/n0	Suriv. time	Inf. tit estimates	n/n0	Suriv. time	Inf. tit estimates	n/n0	Suriv. time	Inf. tit estimates	n/n0	Suriv. time	Inf. tit estimates	n/n0	Suriv time
Frontal cortex	6/6	281 ± 14	10^6.74^ (10^6.27^, 10^7.2^)	6/6	289 ± 15	10^6.54^ (10^6.08^, 10^7.01^)	6/6	295 ± 17	10^6.43^ (10^5.96^, 10^6.89^)	6/6	309 ± 19	10^6.11^ (10^5.64^, 10^6.57^)	0/6	> 600	–	0/6	> 700
Cervical Lymph node	6/6	406 ± 25	10^4^ (10^3.6^, 10^4.5^)	5/6	493 ± 52	10^2.5^ (10^2.1^, 10^2.9^)	6/6	376 ± 18	10^4.6^ (10^4.2^, 10^5.1^)	6/6	422 ± 10	10^3.7^ (10^3.3^, 10^4.1^)	6/6	390 ± 49	10^4.3^ (10^3.9^, 10^4.8^)	0/6	> 700
Spleen	6/6	344 ± 14	10^5.3^ (10^4.9^, 10^5.8^)	6/6	351 ± 3	10^5.2^ (10^4.7^, 10^5.7^)	5/5	337 ± 41	10^5.5^ (10^5^, 10^6^)	6/6	438 ± 35	10^3^ (10^3^, 10^3.4^)	6/6	485 ± 49	10^2.6^ (10^2.3^, 10^3^)	0/6	> 700
Thymus	6/6	513 ± 43	10^2.2^ (10^1.8^, 10^2.6^)	4/7	539 ± 54	10^1.8^ (10^1.2^, 10^2.2^)	NA			5/6	517 ± 40	10^2.2^ (10^1.8^, 10^2.6^)	1/6	560	10^1.3^ (10^0.2^, 10^2.2^)	0/6	> 700
Lung	2/6	601, 601^†^	10^0.55^ (10^–0.1^, 10^1.2^)	0/6	> 600	–	6/6	495 ± 29	10^2.5^ (10^2.1^, 10^2.9^)	0/6	> 600	–	5/6	537 ± 62	10^1.3^ (10^0.9^, 10^1.8^)	0/6	> 700
Heart	2/6	543, 600	10^1.2^ (10^0.4^, 10^1.9^)	4/6	492 ± 36	10^2.4^ (10^2^, 10^2.9^)	1/6	586	10^0.9^ (10^–0.2^, 10^1.9^)	6/6	493 ± 18	10^2.5^ (10^2.1^, 10^2.9^)	2/6	504, 542	10^1.9^ (10^1.3^, 10^2.5^)	0/6	> 700
Liver	4/6	528 ± 63	10^1.9^ (10^1.4^, 10^2.4^)	5/6	510 ± 52	10^1.6^ (10^1.2^, 10^1.6^)	6/6	429 ± 35	10^3.5^ (10^3.1^, 10^4^)	2/6	618, 618^†^	10^0.2^ (10^–0.7^, 10^1^)	0/6	> 600	–	0/6	> 700
Kidney	1/6	531	10^1.8^ (10^0.8^, 10^2.5^)	0/6	> 600	–	4/6	513 ± 51	10^2.2^ (10^1.7^, 10^2.7^)	6/6	498 ± 24	10^2.5^ (10^2.1^, 10^2.8^)	0/6	> 600	–	0/6	> 700
Salivary gland	4/6	553 ± 53	10^1.5^ (10^1^, 10^2^)	0/6	> 600	–	5/6	465 ± 22	10^2.9^ (10^2.5^, 10^3.4^)	4/6	505 ± 27	10^2.3^ (10^1.9^, 10^2.7^)	0/6	> 600	–	0/6	> 700
Pancreas	0/6	> 600	–	1/6	606^†^	10^0.4^ (10^–0.7^, 10^1.5^)	2/6	523, 593	10^1.3^ (10^0.6^, 10^2^)	1/7	627^†^	10^0^ (10^–1.2^, 10^1.1^)	2/6	572, 600	10^0.8^ (10^0^, 10^1.6^)	0/6	> 700
Thyroid	4/6	480 ± 68	10^2.4^ (10^1.9^, 10^2.8^)	1/6	531	10^1.7^ (10^0.7^, 10^2.4^)	1/6	626^†^	10^0^ (10^–0.2^, 10^1.1^)	7/7	518 ± 39	10^2.2^ (10^1.8^, 10^2.5^)	1/6	574	10^1^ (10^–0.1^, 10^2^)	0/6	> 700
Adrenal gland	6/6	443 ± 52	10^3.3^ (10^2.9^, 10^4.5^)	4/6	471 ± 63	10^2.7^ (10^2.3^, 10^3.1^)	3/6	485 ± 38	10^2.6^ (10^2.2^, 10^3.1^)	6/6	423 ± 25	10^3.7^ (10^3.2^, 10^4.1^)	1/6	503	10^1.9^ (10^1.1^, 10^2.6^)	0/6	> 700
Bone marrow	6/6	447 ± 91	10^3^ (10^2.4^, 10^3.5^)	4/6	504 ± 10	10^1.3^ (10^0.8^, 10^1.7^)	5/5	458 ± 37	10^2.1^ (10^1.6^, 10^2.5^)	6/6	373 ± 35	10^3.7^ (10^3.3^, 10^4.2^)	0/6	> 600	–	0/6	> 700
Skeletal muscle	3/6	554 ± 47	10^1.5^ (10^0.8^, 10^2^)	NA		–	6/6	496 ± 31	10^2.5^ (10^2.1^, 10^2.9^)	3/6	547 ± 82	10^2.2^ (10^1.6^, 10^2.6^)	0/6	> 600	–	0/6	> 700
Testis	6/6	458 ± 20	10^3.1^ (10^3.5^, 10^3.5^)	5/6	488 ± 53	10^2.8^ (10^2.4^, 10^3.2^)	6/6	467 ± 24	10^2.9^ (10^2.5^, 10^3.3^)	–	–	–	NA		–	–	–
Ovary			–			–			–	6/6	511 ± 44	10^2.3^ (10^1.9^, 10^2.6^)				0/6	> 700

Tg Bov mice were inoculated with homogenates (10% weight / volume) of tissues that had been collected post mortem from symptomatic vCJD patients (vCJD-1 to vCJD-4) or an asymptomatic vCJD-infected individual (vCJD-AS). vCJD-1 to vCJD-4 were homozygous for methionine at codon 129 of the *PRNP* gene. Patient vCJD-AS was heterozygous (methionine/valine) at codon 129 of the *PRNP* gene.. Tissues from a methionine at codon 129 individual that was not affected with CJD (negative control) were also inoculated in tg Bov. Mice were euthanized when they showed clinical signs of prion infection or after 600–700 days post-inoculation. Mice were considered prion infected when abnormal PrP deposition was detected in brain by Western Blot (PrP^res^ -antibody Sha31 epitope YEDRYYRE). Survival times are presented as mean ± SD except when less than three animals were positive. In that case individual survival time of positive animals are reported. Infectious prion titres were estimated by using the method of Arnold et al. [4]. The method uses the probability of survival (attack rate at each dilution) and the individual mouse incubation time at each dilution to estimate infectious load. Infectious titres are given as estimated values

*LD50* 50% lethal dose with lower and higher boundaries of the CI95%, *AS* asymptomatic, *Suriv*. survival, *Inf* infectious, *n* number of abnormal, *PrP* positive mice by western blot, *n0* number of inoculated mice, *NA* not available, *PrP* prion protein, *tit* titre, *vCJD* clinical variant Creutzfeldt-Jakob disease

In all four vCJD affected patients, the inoculation of frontal cortex homogenate resulted in a 100% attack rate disease transmission (Table 2). Only 5 out of the 54 peripheral tissues samples failed to transmit disease in tgBov. Each of the 14 different categories of peripheral tissues caused, at variable extent, occurrence of clinical TSE in tgBov. Based on these transmission results (positive versus absence of transmission), the pattern of vCJD infectivity in peripheral tissues was relatively similar across the four vCJD patients (Table 2).

No TSE clinical signs or PrP^res^ accumulation in the brain was observed in tgBov inoculated with frontal cortex from the asymptomatic vCJD case (> 650 days post inoculation). 8 out of the 13 inoculated categories of peripheral tissues transmitted a disease (lymphoid organs, lung, heart, pancreas and thyroid) in tgBov (Table 2), which indicated a more restricted distribution of the prion infectivity in the organs of this asymptomatic Met/Val_129_ patient than in the clinically affected Met/Met_129_ vCJD patients.

**Fig. 1:**
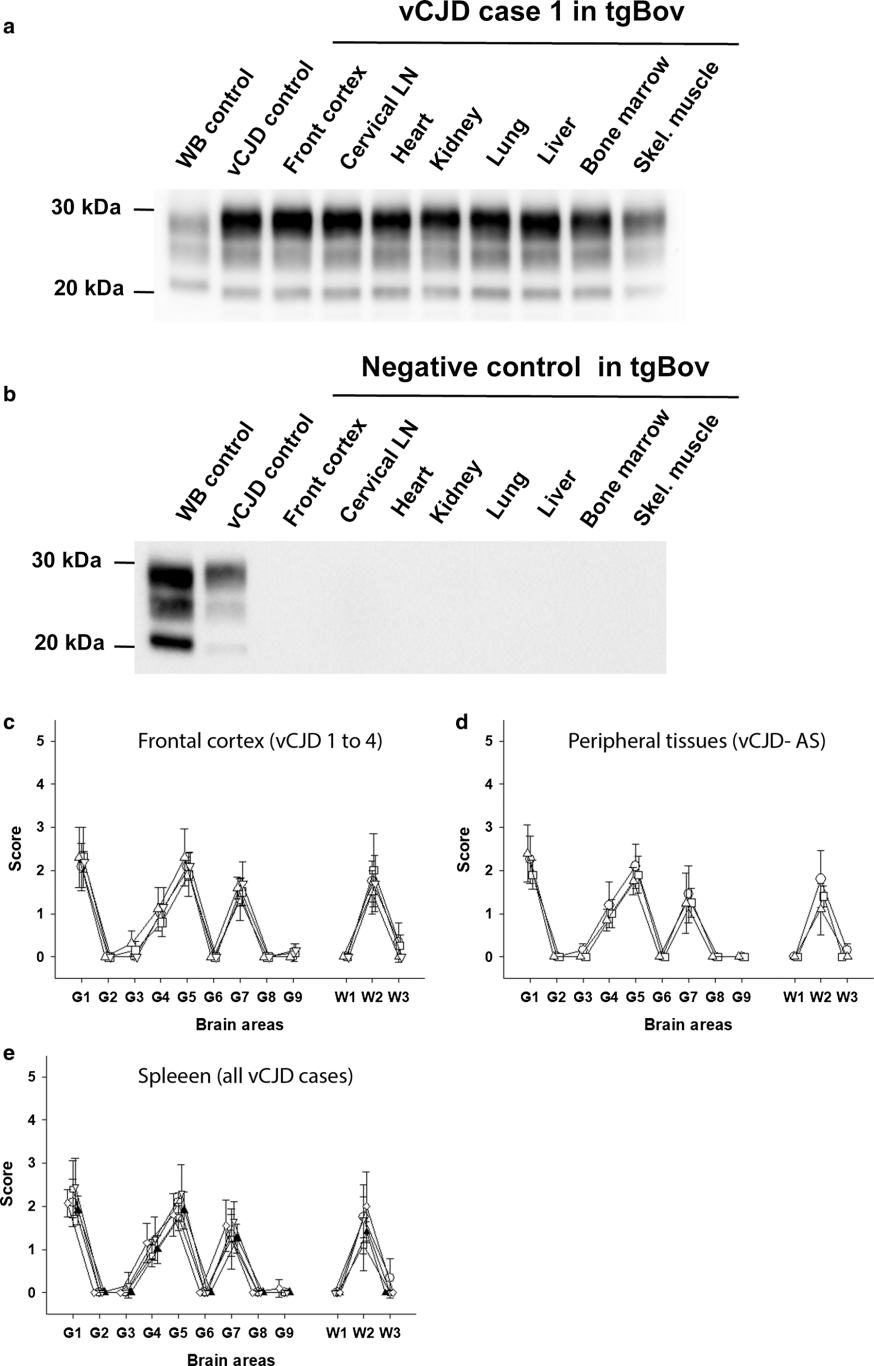
Abnormal PrP and vacuolar lesion profiles in the brain of tgBov mice. Transgenic mice expressing bovine PrP (tgBov) were inoculated with tissues (10% weight/volume homogenates) from 4 vCJD cases (vCJD 1 to 4, homozygous Met_129_), one vCJD asymptomatic case (vCJD-AS, Met/Val 129 heterozygous) and one non CJD control patient (homozygous Met_129_) (Table 1). Mice were euthanized when they showed clinical signs of infection or at the end of their natural life-span (600–700 dpi) and their brains were systematically collected. Western-blot and PrP^res^ immunodetection in the brain of tgBov mice inoculated with tissue homogenates **a** from case vCJD-1 and **b** the non CJD control case. PrP^res^ Western blots were performed using the Sha31 monoclonal antibody (epitope: _145_YEDRYYRE_152_ of the human-PrP). On each gel (i) a scrapie in sheep isolate (WB cont.) and a vCJD reference isolate were used as controls. Vacuolar lesion profiles in the mice of tgBov mice inoculated with, **c** frontal cortex from vCJD-1 (empty circle), vCJD-2 (empty inverted triangle), vCJD-3 (empty triangle), vCJD-4 (empty square) and **d** spleen from vCJD-1 (empty circle), vCJD-2 (empty inverted triangle), vCJD-3 (empty diamond), vCJD-4 (empty square) and vCJD-AS (filled triangle). **e** Lesion profiles were established in tgBov mice that were scored PrP^res^ positive by western blot following the inoculation with the vCJD-AS case cervical lymph node (empty circle), lung (empty triangle), and spleen (empty square). Lesions profiles were established (using at least 5 individual mice) following the standard method described by Fraser and Dickinson in nine grey matter brain areas (G1 to G9) and three white matter brain areas (W1 to W3) [21]

The PrP^res^ Western blot profile and the vacuolar lesions profiles observed in mice inoculated with peripheral tissues from both clinical vCJD and asymptomatic patients were identical to those observed in tgBov mice inoculated with the brain of the vCJD affected patients (Fig. 1).

No transmission was observed in tgBov mice that received frontal cortex and peripheral tissues homogenates from a non CJD control patient (Met_129_ homozygous) (Table 2, Fig. 1).

### sCJD transmission

Five sCJD patients were selected on the basis of their clinico-pathological features, genotype at codon 129 of the *PRNP* gene (Met_129_ homozygous) and PrP^res^ Western Blot type 1 profile in the brain (MM1 sCJD cases) (Table 1). This is the commonest subtype of sCJD. A panel of 15 peripheral tissues collected from these MM1 sCJD patients was constituted. This panel matched the one investigated in vCJD affected patients (Table 3).

**Table 3:** Brain and peripheral tissue samples bioassay results in Methionine 129 Human PrP–expressing mice (tgMet) for sCJD patients

	sCJD 1 (96–1999)			sCJD 2 (21/2006)			sCJD 3 (02/83)			sCJD 4 (03/20)			sCJD 5 (31/2009)			Negative control	
Tissue	n/n0	Suriv. time	Inf. tit estimates	n/n0	Suriv time	Inf. tit estimates	n/n0	Suriv time	Inf. tit estimates	n/n0	Suriv time	Inf. tit estimates	n/n0	Suriv time	Inf. tit estimates	n/n0	Suriv time
Frontal cortex	6/6	215 ± 8	10^7.2^ (10^6.7^, 10^7.7^)	5/5	201 ± 6	10^7.8^ (10^7.2^, 10^8.3^)	5/5	236 ± 9	10^6.4^ (10^5.9^, 10^6.9^)	6/6	225 ± 14	10^5.8^ (10^5.4^, 10^6.3^)	6/6	219 ± 18	10^6.1^ (10^5.6^,10^6.6^)	0/6	> 750
Cervical Lymph node	0/6	> 750	–	6/6	335 ± 47	10^3^ (10^2.7^, 10^3.4^)	0/6	> 750	–	0/6	> 750	–	2/6	294,326	10^2.9^ (10^2.4^,10^3.4^)	0/6	> 750
Tonsil	0/6	> 750	–	6/6	298 ± 21	10^4.2^ (10^3.8^, 10^3.7^)	0/6	> 750	–	1/6	380	10^2^ (10^1.2^, 10^2.6^)	1/6	486	10^0.1^ (10^–0.9^,10^1.2^)	0/6	> 750
Spleen	NA		–	1/5	318 ± 21	10^2.5^ (10^2.1^, 10^2.9^)	0/6	> 750	–	0/6	> 750	–	0/6	> 750	–	0/6	> 750
Thymus	NA		–	NA		–	NA		–	NA		–	4/6	347 ± 19	10^2.2^ (10^1.7^,10^2.5^)	0/6	> 750
Lung	5/5	321 ± 21	10^3.3^ (10^2.9^, 10^3.8^)	0/6	> 750	–	3/6	370 ± 98	10^0.5^ (10^–0.2^, 10^1.2^)	0/6	> 750	–	0/6	> 750	–	0/6	> 750
Heart	5/6	329 ± 43	10^2.9^ (10^2.6^, 10^3.3^)	5/6	381 ± 58	10^2.2^ (10^1.9^, 10^2.5^)	0/6	> 750	–	0/6	> 750	–	1/6	538	10^–3.4^ (10^–4.5^,10^–2.2^)	0/6	> 750
Liver	0/6	> 750	–	0/6	> 600	–	0/6	> 750	–	0/6	> 750	–	0/6	> 750	–	0/6	> 750
Kidney	1/6	403	10^1.6^ (10^0.5^, 10^2.3^)	2/6	302,393	10^2.7^ (10^2.2^, 10^3.3^)	0/6	> 750	–	1/6	381	10^1.9^ (10^1.1^, 10^2.4^)	0/6	> 750	–	0/6	> 750
Salivary gland	5/6	387 ± 61	10^2.1^ (10^1.7^, 10^2.5^)	4/6	367 ± 60	10^2.1^ (10^1.6^, 10^2.6^)	0/6	> 750	–	0/6	> 750	–	2/6	730,732	10^–0.52^ (10^–1.8^,10^1.1^)	0/6	> 750
Pancreas	0/6	> 750	–	2/6	291,331	10^2.8^ (10^2.1^, 10^2.6^)	0/6	> 750	–	5/6	348 ± 63	10^2.7^ (10^2.4^, 10^3^)	1/6	477	10^0.1^ (10^–1.2^,10^0.4^)	0/6	> 750
Thyroid	NA		–	0/6	> 750	–	0/6	> 750	–	1/6	414	10^1.4^ (10^0.2^, 10^2.1^)	0/6	> 750	–	0/6	> 750
Adrenal gland	3/6	526 ± 63	10^–0.6^ (10^–1.1^, 10^0.2^)	6/6	301 ± 9	10^4^ (10^3.6^, 10^4.5^)	5/6	333 ± 32	10^2.8^ (10^2.5^, 10^3.2^)	2/6	345,348	10^2.2^ (10^1.8^, 10^2.6^)	3/6	419 ± 89	10^0.1^ (10^–0.9^,10^1.2^)	0/6	> 750
Bone marrow	6/6	264 ± 11	10^4.4^ (10^3.9^,10^4.9^)	6/6	359 ± 38	10^1.6^ (10^1.3^, 10^1.9^)	3/6	409 ± 18	10^0.2^ (10^0^, 10^0.9^)	6/6	311 ± 19	10^2.7^ (10^2.2^, 10^3.1^)	6/6	316 ± 18	10^2.5^ (10^2.1^,10^2.9^)	0/6	> 750
Skeletal muscle	1/6	485	10^–1.4^ (10^–2.5^, 10^–0.2^)	5/5	304 ± 32	10^3.9^ (10^3.4^, 10^4.4^)	3/6	484 ± 81	10^–0.2^ (10^–0.8^, 10^0.5^)	0/6	> 750	–	0/6	> 750	–	0/6	> 750
Testis							NA										
Ovary	0/6	> 750	–	NA						NA			0/6	> 750	–	0/6	> 750

Tg Met mice were inoculated with homogenates (10% weight / volume) of tissues that had been collected post mortem from symptomatic sCJD patients (sCJD-1 to sCJD-5). All the sCJD patients were homozygous for methionine at codon 129 of the *PRNP* gene and displayed a type 1 abnormal PrP profile in their brain. Tissues from a methionine at codon 129 individual that was not affected with CJD (negative control) were also inoculated in tg Met. Mice were killed when they showed clinical signs of prion infection or after 750-days post-inoculation. Mice were considered prion infected when abnormal PrP deposition was detected in brain by Western Blot (PrP^res^ -antibody Sha31 epitope YEDRYYRE). Survival times are presented as mean ± SD except when less than three animals were positive. In that case individual survival time of positive animals are reported. Infectious prion titres were estimated by using the method of Arnold et al. [4]. The method uses the probability of survival (attack rate at each dilution) and the individual mouse incubation time at each dilution to estimate infectious load. Infectious titres are given as estimated values

*LD50* 50% lethal dose with lower and higher boundaries of the CI95%, *Suriv*. survival, *Inf *infectious, *n* number of abnormal, *PrP* positive mice by western blot, *n0* number of inoculated mice; *NA* not available, *PrP* prion protein, *sCJD* sporadic Creutzfeldt–Jakob disease; *tit* titre

Each sample (10% tissue homogenates) was inoculated by the intracerebral route (IC) to Met_129_ human PrP expressing mice (tgMet, *n* = 6 per sample, 20µL per mouse), a mouse model that we already used to detect and quantify prion infectivity in MM1 sCJD patients [32].

**Fig. 2:**
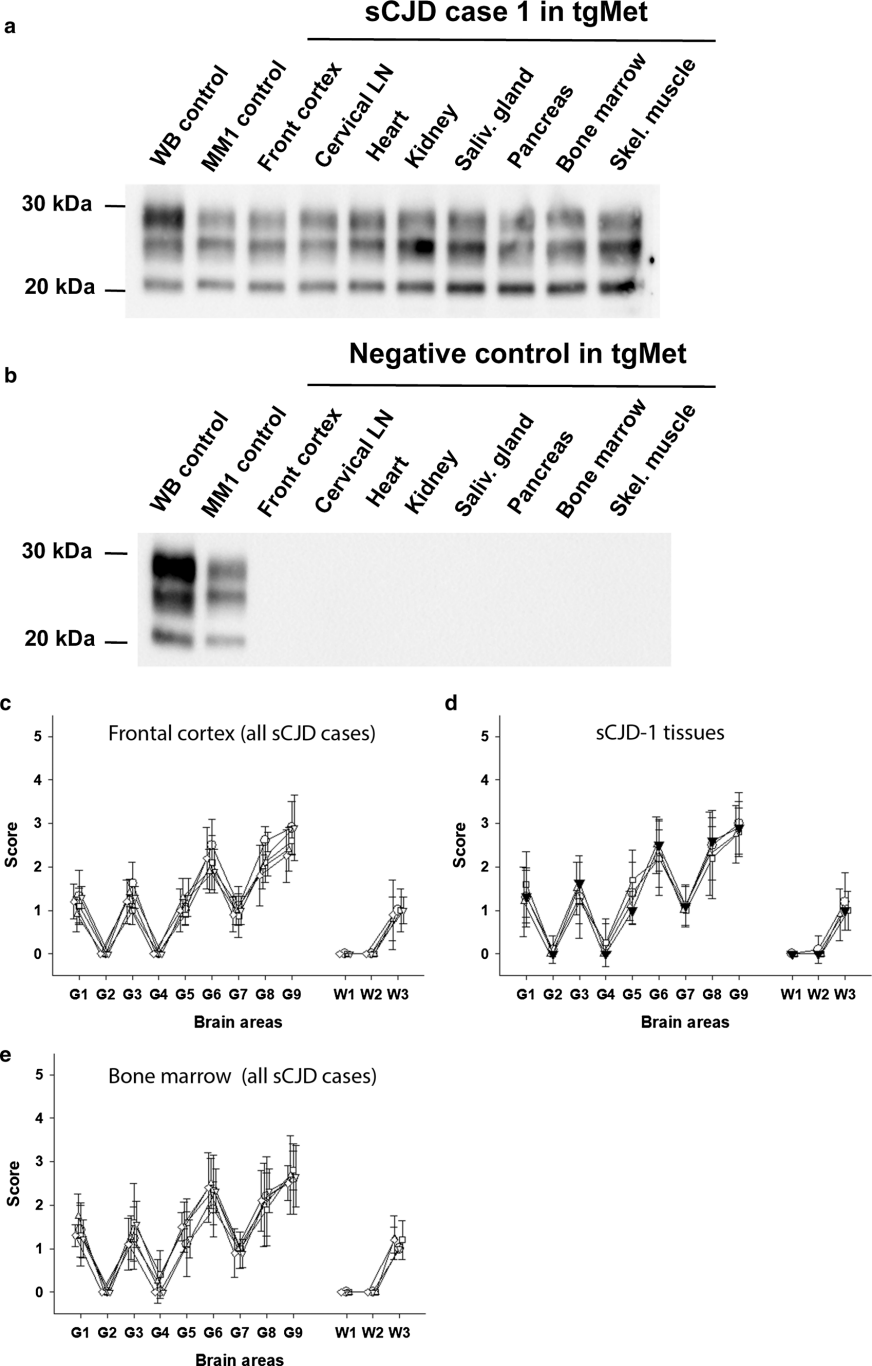
Abnormal PrP and vacuolar lesion profiles in the brain of tgMet mice. Transgenic mice expressing the methionine 129 (TgMet) variant of the human-*PRNP* were inoculated with tissues (10% weight/volume homogenates) from 5 MM1 sCJD cases and one homozygous Met_129_ non CJD control case (Table 1). Mice were euthanized when they showed clinical signs of infection or at the end of their natural life-span (> 750 dpi) and their brains were systematically collected. Western-blot and PrP^res^ immunodetection in the brain of tgMet mice inoculated with tissues homogenates **a** from case sCJD-1 and **b** the non CJD control case. PrP^res^ Western blots were performed using the Sha31 monoclonal antibody (epitope: _145_YEDRYYRE_152_ of the human-PrP). On each gel (i) a scrapie in sheep isolate (WB cont.) and a MM1 sCJD reference isolate were used as controls. Vacuolar lesion profiles in the mice of tgMet mice inoculated with, **c** frontal cortex and bone marrow **d** from sCJD-1 (empty circle), sCJD-2 (empty inverted triangle), sCJD-3 (empty triangle), sCJD-4 (empty square). **e** Similarly, lesion profiles were established in tgMet mice that were scored PrP^res^ positive by western blot following the inoculation with the sCJD-1 case frontal cortex (filled inverted triangle), lung (empty circle), heart (empty triangle), and salivary gland (empty square). Lesions profiles were established (using at least 5 individual mice), following the standard method described by Fraser and Dickinson in nine grey matter brain areas (G1 to G9) and three white matter brain areas (W1 to W3) [21]

The inoculation of 10% frontal cortex homogenates from the sCJD MM1 patients in tgMet (IC route, 6 mice, 20µL per mouse) resulted in a clinical TSE with mean survival times comprised between 200 and 240 days (Table 3). The PrP^res^ WB profile and the vacuolar lesion profile in the brain of the tgMet indicated that a same prion strain was present in the frontal cortex of these five sCJD patients (Fig. 2).

Unexpectedly, in the majority of the cases, the inoculation in tgMet of the peripheral tissues from the same MM1 sCJD patients resulted in positive transmission. TgMet inoculation revealed the presence of prion infectivity in all the different categories of peripheral tissues except liver and gonads (Table 3).

However, in contrast with vCJD, the bioassay results (positive versus absence of transmission) indicated that the prion infectivity distribution pattern in peripheral tissues strongly differed between the five affected MM1 sCJD patients. For instance, in sCJD case 2, positive transmissions were observed in tgMet inoculated with 10 out of the 13 tested peripheral tissues including lymphoid tissues, salivary glands, kidney, heart and pancreas. In contrast, in sCJD case 3, positive transmissions were only observed for 4 out of the same 13 tissues (lung, adrenal gland, bone marrow and skeletal muscle), with no disease transmission resulting from inoculation of lymphoid tissues, salivary gland, kidney, heart or pancreas (Table 3).

Interestingly, bioassay of the lymphoid tissues (spleen, cervical lymph node and tonsil) resulted in a disease transmission in only 3 out of the 5 sCJD cases (cases 2, 4 and 5). These results support the contention that in MM1 sCJD patients, the presence / absence of infectivity in the lymphoid organs is apparently not a determinant driver of the accumulation of infectivity in the other categories of peripheral tissues.

Despite the differences in bioassay transmission patterns, the PrP^res^ Western blot profile and the vacuolar lesions profiles overserved in tgMet mice inoculated with MM1 sCJD peripheral tissues and frontal cortex brain homogenates were identical (Fig. 2), indicating that the same prion strain was present in the peripheral tissues and the CNS of the five MM1 sCJD patients.

No transmission or PrP^res^ accumulation was observed in tgMet mice inoculated with peripheral tissues from the non CJD control patient (Table 3, Fig. 2).

### Infectivity titres estimates

In order to estimate the infectivity levels in the vCJD and MM1 sCJD patients’ tissues, we applied the method described by Arnold et al. [4]. This approach uses both the probability of survival (attack rate at each dilution) and the individual mouse survival time at each dilution. The relationship between the titre of inoculum and the probability of infection and the length of the survival times were derived from data corresponding to endpoint titration of a vCJD and a MM1 reference isolate in tgBov and tgMet mice, respectively [17, 32]. A normal distribution for the relationship between dose and survival time was assumed and the probability of infection versus dose was assumed to follow a logistic regression curve (supplementary Fig. 1).

Using this approach, the infectious titre in the frontal cortex of the four vCJD patients was estimated to range between 10^6.11^ and 10^6.74^ ID_50_ IC in tgBov per gram of tissue (Table 2).

**Fig. 3:**
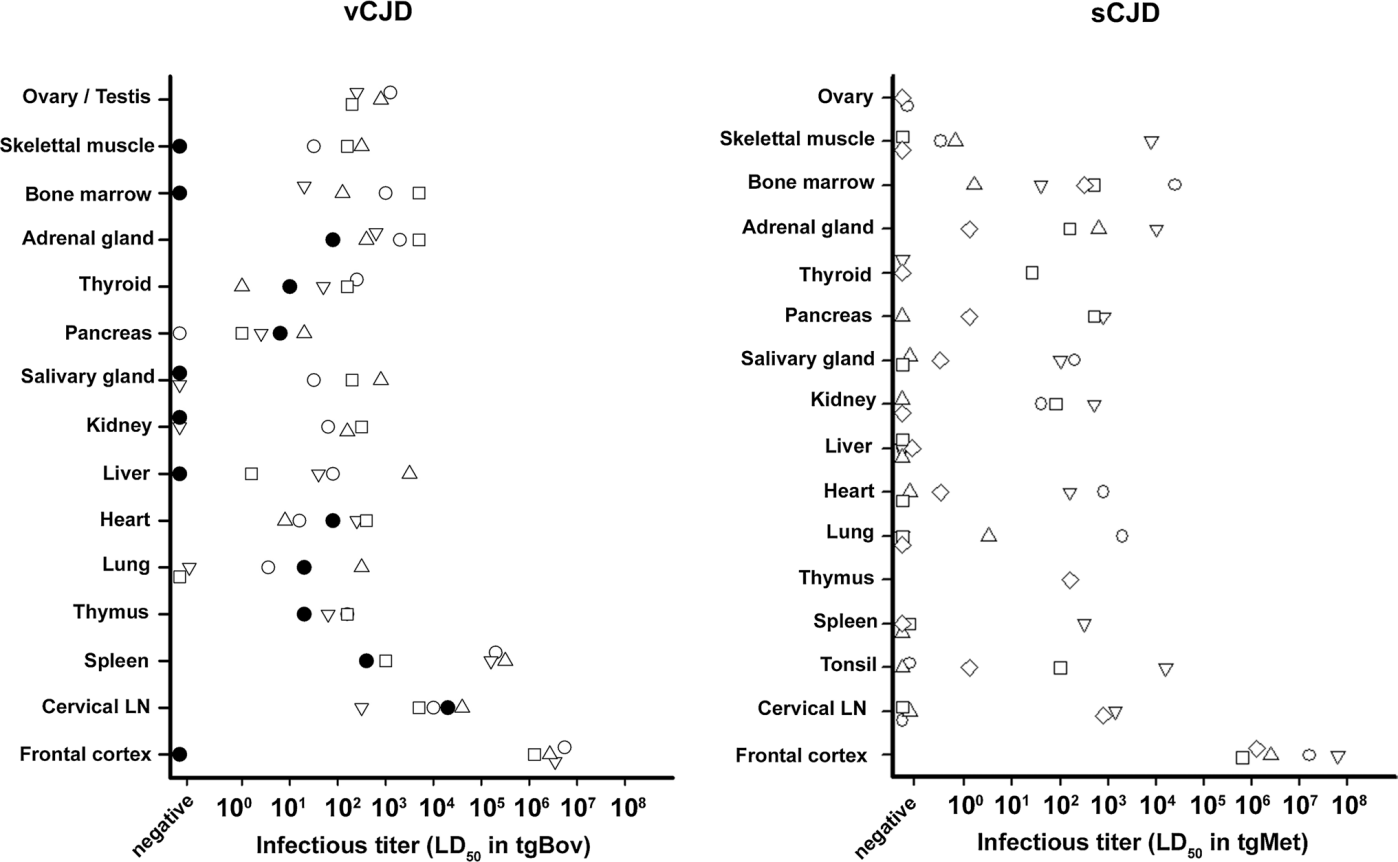
infectivity levels in peripheral tissues and frontal cortex of vCJD and sCJD patients. Cases vCJD-1 (empty circle), vCJD-2 (empty inverted triangle), vCJD-3 (empty triangle) and vCJD-4 (empty square) died with clinical vCJD. These four patients were Methionine homozygotes at codon 129 of the *PRNP* gene. Patient vCJD-AS (filled circle) died at an asymptomatic stage of the disease. This patient was heterozygous Methionine/Valine at codon 129 of the *PRNP* gene. Cases sCJD-1 (empty circle), sCJD-2 (empty inverted triangle), sCJD-3 (empty triangle), sCJD-4 (empty square) and sCJD-5 (empty diamond) died with clinical sCJD. All five were Methionine homozygotes at codon 129 of the *PPNP* gene. 10% weight/volume tissues homogenates (frontal cortex and peripheral tissues) from vCJD and sCJD cases were prepared and inoculated in bovine PrP (tgBov) and Met129 human PrP expressing mice respectively. Mice were euthanized when they showed clinical signs of infection or after 600 dpi. Mice were considered infected when abnormal PrP deposition was detected in brain by western blot using the Sha31 monoclonal antibody: epitope amino acids 145–152 (YEDRYYRE) of the sheep PrP sequence. Infectious prion titres were estimated using the method developed by Arnold et al*.* [4]. The model uses both the probability of survival (attack rate at each dilution) and the individual mouse survival times at each dilution to estimate the infectious load

The estimated infectivity levels in secondary lymphoid tissues (spleen and cervical lymph node) were 1 to 4 log10 lower than in the frontal cortex of the same patient. Infectivity levels in the other categories of peripheral tissues were 2.5 to 6 log10 lower than in the frontal cortex (Table 2, Fig. 3).

Strikingly, in some of the vCJD affected patients, infectivity levels in heart (vCJD-3 and 4), kidney (vCJD-3 and 4), lung(vCJD-3), salivary gland (vCJD-3 and 4) or thyroid (vCJD-4) were only 1 to 2 log10 lower than in the spleen and/or cervical lymph node (Table 2, Fig. 3).

As already stated, the distribution of the prion in the peripheral tissue of the vCJD asymptomatic case was more restricted than in vCJD affected patients (Fig. 3). However, when positive, the peripheral tissues from the asymptomatic patient displayed similar infectivity levels to those observed in the vCJD patients at the clinical stage of the disease (Table 2, Fig. 3).

In the MM1 sCJD patients, the estimated infectivity levels in the frontal cortex varied between 10^5.8^ and 10^7.8^ ID_50_ IC in tgMet/gram (Table 3). Infectivity levels in peripheral tissues that scored positive in bioassay were 2.8 to 8 log10 lower than in the frontal cortex of the same patient (Table 3, Fig. 3). In all five cases, no obvious relationship seemed to exist between the infectivity level in the CNS (frontal cortex) and either the distribution or the levels of sCJD infectivity in their peripheral tissues (Fig. 3).

In the sCJD cases 2, 4 and 5, the lymphoid tissues displayed maximal level of infectivity that were 3–3.5 log10 lower than those observed in the frontal cortex (Table 2, Fig. 3). Strikingly, the infectivity levels associated with some peripheral tissues such as salivary gland (sCJD2), heart (sCJD2), kidney (sCJD 2) or bone marrow (sCJD 5) could be equivalent or even higher than those measured in the lymphoid organs of the same patient (Table 2, Fig. 3).

## Discussion

### vCJD associated risks

Following the emergence of vCJD in the UK in 1996, the presence of abnormal prion protein in the lymphoid tissues of affected patients was rapidly identified [31, 55]. This immediately raised major concerns about the risk of its iatrogenic transmission of the disease (via contaminated surgical instruments and blood transfusion) and led, in many countries, to the implementation of specific preventing against this risk.

Despite a relatively limited number of identified clinical cases (*n* = 231), the most recent epidemiological studies indicated that 1 out 2000 people in the UK could carry the vCJD agent (as judged by the presence of abnormal prion protein detected by immunohistochemistry in lymphoid follicles in the appendix) and that the exposure period to BSE agent in the UK could have largely exceeded the period initially considered to be at risk (i.e. the 1985–1996 period) [22, 23]. Over the 25 years since the emergence of vCJD only five instances that are a likely consequence of iatrogenic vCJD transmission have come to light, all in the UK and all associated with blood and blood-products [36, 37, 46, 47]. No cases of vCJD due to iatrogenic transmission by medical or surgical procedures have been identified, indicating that the preventive measures implemented to mitigate its transmission were effective [26, 39].

The overall picture of the distribution and levels of prion infectivity in the tissues of both vCJD affected and asymptomatic patients that we here report confirm the numerous hypotheses and the mosaic of experimental data that were used to design the infection control measures that were successful in limiting iatrogenic vCJD transmissions. These results also reinforce the fact that vCJD transmission risks have not disappeared since each asymptomatic vCJD-infected individual in a human population will continue to represent a potential source of disease transmission.

### Peripheral tissue infectivity in sCJD

Seminal transmission experiments of peripheral tissues from sCJD cases in primate models failed to detect infectivity in a large selection of peripheral tissues, body fluids and excretions (except in one liver sample) [10]. Abnormal PrP immunodetection techniques (Western blot and immunohistochemistry) also failed to reveal the presence of prion in the peripheral tissues of sCJD patients [27, 28].These findings led to the generally accepted view that prion infectivity in the sCJD remains mostly confined to the CNS.

In 2003, improved Western Blot protocols for PrP^res^ immunodetection revealed the presence of prion in the spleen (10 positive out of 28 cases) and/or the skeletal muscle (8 positive out of 32 cases) [24]. More recently, transmission studies of sCJD tissues in human PrP expressing transgenic mice (using plasma and bone marrow samples) and in vitro amplification of prions in a small number of sCJD peripheral tissues (skin, kidney, lung, adrenal gland) provided further evidence to the view that prions can accumulate in the peripheral tissues of sCJD affected patients [17, 32, 43, 52].

The results that we here report provide unequivocal and definitive evidence of the widespread distribution of the prion infectivity in the peripheral tissues in MM1 sCJD patients.

### Other types of patients

Since our study was restricted to MM1 sCJD cases (the commonest sCJD subtype), additional investigations will be necessary to formally establish that consistent accumulation of prions in peripheral tissues also occurs in patients with other *PRNP* genotypes (Met/Val_129_ and Val/Val_129_) and/or affected with other sCJD prion strains [11]. The presence of abnormal PrP (in spleen and the skeletal muscle) and infectivity (in bone marrow) already identified in MV2 and VV2 sCJD patients indicates that this phenomenon is unlikely to be limited to MM1 sCJD cases [24, 32].

### Variability in sCJD patients

The nature of peripheral tissues that accumulated infectivity in sCJD and vCJD cases were relatively similar. While the patterns of prion distribution and the infectivity levels observed in the peripheral tissues were relatively homogenous in the vCJD cases, particularly in lymphoid tissues, a high degree of variability was observed across the sCJD cases. Brain vacuolar lesion profiling and PrP^res^ WB typing confirmed that the same prion strain was present in the brain and the peripheral tissues of all the sCJD affected patients. This rules out the hypothesis that prion strain(s) difference(s) (between individuals or in tissues from a same individual) could be responsible for the observed variability.

The comparison of prion distribution pattern in sCJD case 2 (20-months clinical phase duration) and sCJD cases 1, 3, 4 and 5 patients (1–4 months clinical phase duration) might suggest that, at first glance, a longer clinical phase duration is likely to be associated with a more widespread distribution of prions in the body tissues. However, even if our study represents an unprecedented effort for characterizing CJD agent distribution patterns in the organs of affected patients, the number of sCJD cases that we investigated is too limited to draw definitive conclusions. The characterisation of a larger cohort of sCJD patients will be necessary to establish the relationship that might exist between the distribution and/or infectivity levels in peripheral tissues and sCJD patient age at clinical onset and the duration of the clinical phase of the illness.

### Iatrogenic transmission of CJD

Several hundred cases of iatrogenic CJD transmission (iCJD) have been reported worldwide, the vast majority of which are likely to represent transmissions from sCJD patients [9]. The principal sources of these outbreaks were intramuscular injections with contaminated human pituitary-derived growth hormone (226 cases) and implantation of dura mater grafts (228 cases) derived from human cadavers with undiagnosed sCJD infections. A small number of cases were apparently also caused by neurosurgery using contaminated neurosurgical instruments and EEG electrodes (6 cases), transplantation of corneal grafts (2 cases) and intramuscular injections with human pituitary-derived gonadotrophic hormone (4 cases) [9].

These cases dramatically illustrate the high resistance of CJD prions to standard medical decontamination procedures and their particular abilities to bind to steel surgical instruments [19, 53]. Survival times in the individuals who were exposed to sCJD agent(s) by the peripheral route could be extremely long and variable; for instance, in patients that received intramuscular injection of contaminated human pituitary-derived growth hormone, the onset of clinical signs and symptoms of iatrogenic CJD could be observed between 4 and 42 years after the treatment [9].

In a context where sCJD infectivity is apparently limited to the CNS, the medical and surgical procedures responsible for iatrogenic transmission of the disease remain relatively limited, the overall risks for sCJD iatrogenic transmission are now considered to be remote since most of these established routes of transmission be avoided (e.g. the use of human pituitary-derived hormones and human dura mater grafts) [9].

Our detection of low levels of sCJD infectivity in non-CNS tissues such as lung, heart, muscle or even salivary gland was unexpected. We next reviewed the medical histories for the sCJD patients that we studied (Table 1), which revealed surgical procedures (s-CJD-2 and 4) and/or invasive medical examinations (polypectomy under colonoscopy sCJD-5) only few years before the clinical disease in these patients. Some years ago, the presence of infectivity in the plasma and the detection of abnormal prion protein in the urine of sCJD patients raised concerns about the risks of sCJD transmission by blood transfusion and plasma/urine-derived medical products [17, 38]. The detection, in our study, of infectivity in the bone marrow and the kidney of sCJD patients further reinforces these concerns.

Prion titres as measured by intracerebral inoculation in PrP over-expressing transgenic mice models can provide us with estimates of the relative infectivity levels present in the CNS and peripheral tissues of affected patients. This approach conforms to the current gold standard for quantification of prions. However, in a context where the amount of infectivity that would be necessary to transmit disease to another human remains unknown, the infectivity titre estimates (as established following intracerebral inoculation in a reporter animal model) cannot be used to directly infer transmission risks to patients. Other factors, including the various different potential exposure routes (subcutaneous, intramuscular, etc.) and the decontamination/sterilisation methodologies used on the surgical instruments /materials involved, will also influence the transmission risk analysis [1].

In many industrialized countries, reliable CJD passive surveillance programs have been established for decades. The apparently stable and low prevalence of sCJD cases in these countries bring some reassurance about the low numbers of iatrogenic CJD cases in this century [9], perhaps reflecting the variable and lower prion titres detected in the non-CNS tissues in this study.

Guidelines are in place to mitigate and control the risk of iatrogenic transmission of CJD in a healthcare setting [1, 56]. The procedures for cleaning and decontamination of surgical instruments and/or medical equipment now includes an assessment of their potential contamination by prions. However, prions are notoriously resistant to physico/chemical treatments and decontamination process that would be efficient on these agents remain generally inapplicable to some surgical and medical equipment [54], resulting in recommendations to destroy neurosurgical instruments that have been used on the brain of a patient with definite or probable CJD. Furthermore, the presence of dementia or an evolving neurodegenerative disorder in a patient undergoing medical or surgical procedures triggers the use of specific protocols designed to prevent the risk of potential transmission of CJD from the equipment used (surgical tools, endoscopes, etc.). The use of cells/tissues/organs and body fluids from these groups of patients for therapeutic purposes (blood donations, tissue grafts, etc.) is also restricted [1, 56].

When considering the risk of iatrogenic transmission of prion agents from CJD patients, one fundamental question is: How early before the occurrence of clinical signs and symptoms is prion infectivity likely to be present in peripheral solid tissues, blood and urine?

All the samples that we used to establish infectivity levels in tissues were collected post mortem in sCJD and vCJD patients at the terminal stage of the disease. There is clear evidence for the presence of infectivity in the blood and the peripheral tissues years before the clinical onset of vCJD in asymptomatic infected patients [3, 8, 16, 17]. However, it is uncertain that the infectivity levels and distribution in the post mortem peripheral tissues of sCJD and vCJD patients reflect the situation that could be observed at a preclinical or early clinical stage in the same patients. In the absence of tissue samples collected from asymptomatic sCJD patients we are totally lacking in data, therefore trying to elaborate further on this question would be totally speculative.

In several forms of genetic prion disease, for example those associated with the E200K *PRNP* mutation, the clinical disease manifestations, PrP^res^ WB signature and tissue PrP^res^ distributions are similar to that in sCJD [34, 48]. Despite the ethical issues it might raise, the longitudinal collection of blood samples and body fluids, for research purposes, in consenting patients belonging to families affected by these genetic forms of prion diseases (with confirmed mutations of the *PRNP* gene) may represent the only possibility to address this question.

In conclusion, the systematic surveillance of CJD and related epidemiological studies in many countries confirm the decline of cases of iatrogenic CJD due to recognised medical or surgical procedures, such as human dura mater graft surgery or treatment with human pituitary-derived growth hormone. However, they do not exclude the possibility that iatrogenic transmission could at least partly account for some sCJD cases observed in the population, particularly in localized geographic regions with evidence of CJD case clusters [50].

Many uncertainties remain on the early stages of the prion accumulation and infectivity in the peripheral tissues in patients infected with sCJD. However, the results of this study suggest that the iatrogenic transmission risks associated with sCJD peripheral tissues should not be disregarded.

## Supplementary Information

Below is the link to the electronic supplementary material.

Dose-response relationship for the survival time and probability of infection of transgenic mice expressing the methionine 129 (Tg Met) of the human-PrP or the bovine PrP (TgBov). Data corresponding to endpoint titration of a vCJD (Met129 homozygous) reference isolate in tgBov (A) and of a MM1 sCJD reference isolate in tgMet (B) were used to derive the relationship between the prion infectivity titre of the inoculum and the probability of infection and the length of the survival time to estimate prion infectivity levels in these two mouse models following the methodology described by Arnold et al [4]. These data have already been used in previous publications [15, 32] (TIFF 8484 KB)

## Data Availability

All data are presented in the manuscript and the supplementary information. Human biological material used in the study was provided by the National CJD Research & Surveillance Unit Brain and Tissue Bank in Edinburgh, UK, which is part of the Edinburgh Brain and Tissue Bank supported by the Medical Research Council.
