# Supplementary figures and images for: Wide distribution of prion infectivity in the peripheral tissues of vCJD and sCJD patients
Source: Acta Neuropathol. 2021 Feb 2;141(3):383–97. doi: 10.1007/s00401-021-02270-x (PMC7882550; doi:10.1007/s00401-021-02270-x)

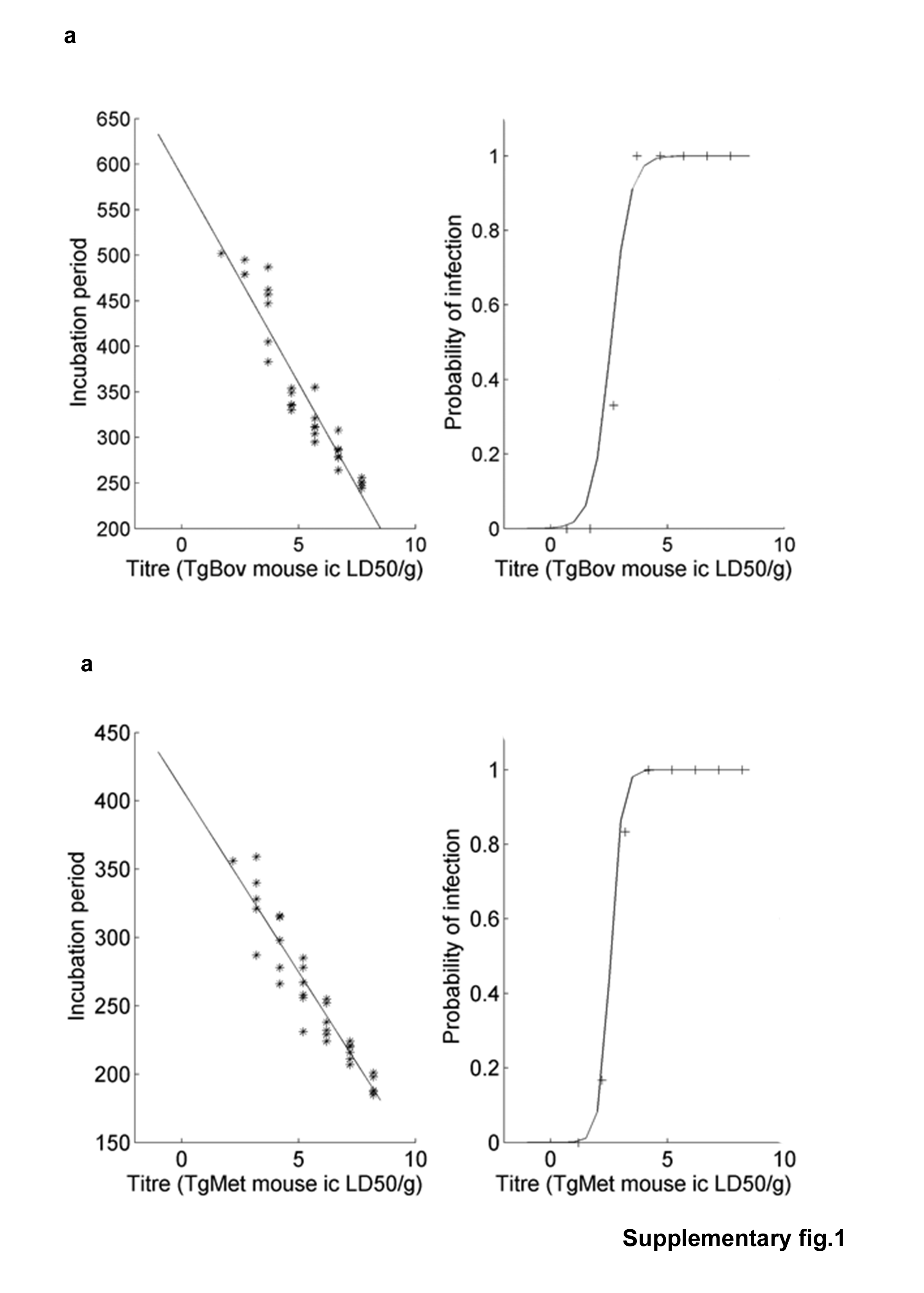

Supplement: Supplementary file 1 — Dose-response relationship for the survival time and probability of infection of transgenic mice expressing the methionine 129 (Tg Met) of the human-PrP or the bovine PrP (TgBov). Data corresponding to endpoint titration of a vCJD (Met129 homozygous) reference isolate in tgBov (A) and of a MM1 sCJD reference isolate in tgMet (B) were used to derive the relationship between the prion infectivity titre of the inoculum and the probability of infection and the length of the survival time to estimate prion infectivity levels in these two mouse models following the methodology described by Arnold et al [4]. These data have already been used in previous publications [15, 32] (TIFF 8484 KB) [file 401_2021_2270_MOESM1_ESM.tif]
